# Assessment of the Nasopalatine Canal Length and Shape Using Cone-Beam Computed Tomography: A Retrospective Morphometric Study

**DOI:** 10.3390/diagnostics14100973

**Published:** 2024-05-07

**Authors:** Cristalle Soman

**Affiliations:** Department of Oral Maxillofacial Surgery and Diagnostic Sciences, College of Medicine and Dentistry, Riyadh Elm University, Riyadh 12734, Saudi Arabia; cristalle.soman@riyadh.edu.sa

**Keywords:** nasopalatine canal, cone-beam computed tomography, incisive canal, implant complications, nasopalatine nerve

## Abstract

The anatomical position of the nasopalatine canal in the anterior maxilla makes it one of the most important vital structures in the region. Surgical and implant procedures in this area require local anesthesia to be administered. It is, therefore, important to morphologically assess the length and shape of the nasopalatine canal for performing surgical procedures with more accuracy in this area. Cone-Beam Computed tomography scans were scrutinized using inclusion criteria of age 18 years and above, absence of any pathological lesions/fracture/surgery in the nasopalatine area, absence of orthodontic treatment or maxillary jaw correction surgeries, and exclusion criteria including CBCT scans with artifacts or error s in the area of interest, anterior implants, absence of bone diseases, trauma, surgeries, and impactions in the area of interest. A total of 360 scans were analyzed for the length and shape of the nasopalatine canals. The results of the study showed that the mean nasopalatine canal length was 12.51 mm. The hourglass shape of the canal was most common and had the highest representation in both genders, with male 80.62% and female 87.01%. A statistically significant difference (*p* < 0.001) was noted in nasopalatine canal length between males and females. The study provides insight into the significant association of gender with the canal’s shape and length of the canal. The length of the canal does not influence with age. These parameters are helpful for surgical planning and interventions in the anterior maxillary region.

## 1. Introduction

The maxilla specifically have multiple foramina and canals, and oral and maxillofacial surgeons have to be cautious while delivering local anesthesia to the area to reduce bleeding and performing different surgical procedures in these areas, such as dental implant placement, orthognathic surgeries involving maxilla, Le Fort I osteotomies, sinus surgeries, and assessment of perineural spread of malignant lesions in palate and occupying lesions (cyst and benign tumors) [1]. 

The anterior maxilla has the nasopalatine canal (NPC) and it contains the neurovascular bundle supplying the anterior maxilla. The anterior segment of the maxilla is also one of the most common parts of the maxilla, which has a tendency towards trauma, bone or tooth loss, pathologies, and anterior implant placements. The above factors can affect the NPC [2,3,4,5,6,7]. 

The trend towards immediate implant is also on the rise. The increase in length of the NPC can also pose risk for neurovascular injuries during implant placement. Nonetheless, maxillary atrophy post-extraction tends for NPC to enlarge up to 32%, occupying around 58% of maxillary alveolar bone width [3]. The nasopalatine canal is a vital structure to be evaluated in various surgical procedures planned in the premaxillary area, such as implant placement, evaluation and post-surgical assessment of pathologies involving the NPC (cyst, benign, and malignant lesions), and evaluation for pre- and post-bone augmentation [1,2,3,4,5,6,7]. 

Morphometric evaluation of NPC has clinical significance in dentistry. Changes in the parameters of NPC dimension or a bulge may indicate fracture involving the dentoalveolar region. During implant planning, safe margins from the NPC should be planned for preventing complications. Breaching these safe margins can lead to hemorrhage, neurosensory disturbances, and implant failure due to lack of osteointegration, and induce the formation of nasopalatine duct cyst [5]. Hence, various studies have been conducted to evaluate the nasopalatine canal region [1,2,3,4,5,6,7]. 

Morphometric analysis of the NPC using two-dimensional imaging provides limited information [3]. Numerous classifications for NPC using various imaging modalities have also been studied over time which indicate both significant differences and similarities in the shapes of the NPC among diverse geographical regions. Studies using three-dimensional cone-beam computed tomography (CBCT) provide detailed three-dimensional information and have been used to evaluate the nasopalatine region based on various planes of the CBCT. Anatomical variations in the nasopalatine canal region pave the way for studies in different populations with varying methodologies [1,2]. The results of many studies are hard to compare due to the demographic data inconsistency in the population as well [3,4,5,6,7]. 

Limited data is available on the correlation of demographic factors on the shape and length of the canal in the Saudi population, and previous studies have also pointed to morphological variations in the region of the NPC. Considering the above factors, there is a need for comprehensive knowledge of the shape and length of the NPC in the Saudi population, which is the rationale of the present study. Thus, the study aimed to analyze the nasopalatine canal length and shape in the Saudi population using cone-beam computed tomography.

## 2. Materials and Methods

**Figure 1:**
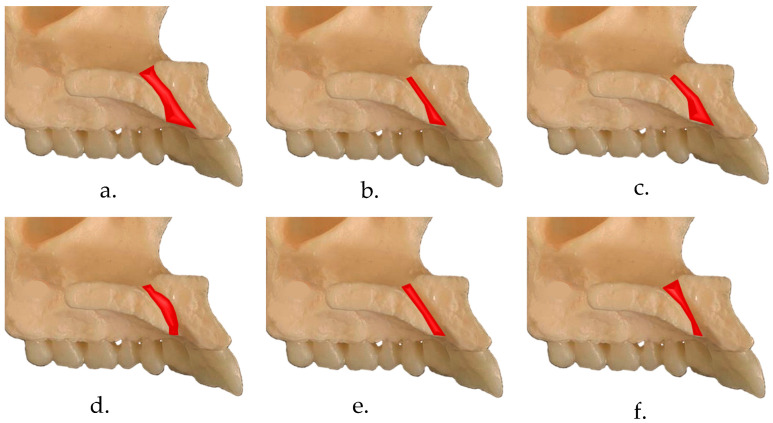
Pictorial representation of the shapes of Nasopalatine canal in the sagittal plane: (**a**) Hourglass shape, (**b**) Cone-shape, (**c**) Funnel shape, (**d**) Banana shape, (**e**) Cylindrical shape, (**f**) Reverse Cone shape.

**Figure 2:**
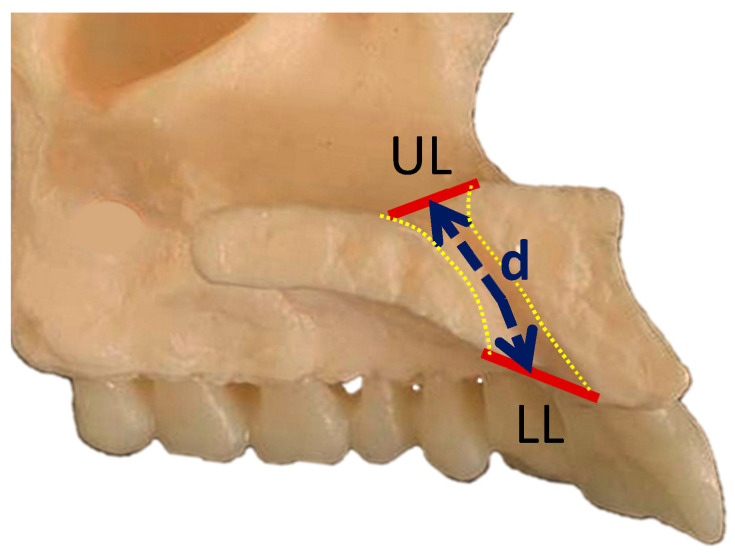
Distance of the nasopalatine canal (d), calculated from the upper limit (UL) of the nasopalatine canal to the lower limit (LL) of the nasopalatine canal.

The shape of NPC was evaluated using multiplanar reformatted views in sagittal sections and categorized into six groups—hourglass, cone, reverse cone, funnel, banana, and cylindrical, as classified by Bahsi et al. [4] and Mardinger et al. [8] (Figure 1). Based on the classification, canal length was assessed in the sagittal section as in the previous study by Milanovic et al. [5]. The length of the course of the nasopalatine canal was measured as the distance between the upper limit set as opening at the nasal level and the lower limit at the level of the hard palate. The cylindrical canal measurement was recorded as a straight-line distance between the upper and lower limits. In the case of all other canal shapes, the measurement was taken along the midplanes of the inclines of the anterior and posterior walls of the canals (Figure 2).

All CBCT scans were collected for five years, from 2016 to 2020, with an estimated minimum sample of 182 scans required for conducting the study, consistent with the previous study [6]. The study was registered with the university ethical review committee with the IRB approval number FRP/2021/440/720/702. Retrospective CBCT scan data was screened according to the eligibility criteria with inclusion criteria of age 18 years and above, absence of any pathological lesions/fracture/surgery in the nasopalatine area, and absence of orthodontic treatment or maxillary jaw correction surgeries, and exclusion criteria included CBCT scans with artifacts or errors in the area of interest, anterior implants, absence of bone diseases, trauma, surgeries, and impactions in the area of interest. All the acquired CBCT scans were referred for multiple reasons, including treatment for impact teeth, periodontal evaluation, and posterior teeth evaluation. Images were analyzed using Galileos viewer I (version 1.9.4497.23802, 2006–2011, Sirona dental system (Charlotte, NC, USA), with a resolution of 287 μm and voxel size of 0.15 mm. A single examiner carried out all measurements and the mean of three readings was considered for each measurement to avoid investigator bias.

Data were entered in Excel and the analysis was performed in STATA v17. Quantitative variables like age and nasopalatine canal (NPC) length were summarized as mean with standard deviation (SD) based on the data’s normality, which was checked using the Kolmogorov–Smirnov test. 

The mean nasopalatine canal (NPC) length between the age category categories was compared using one-way Analysis of Variance (ANOVA). The mean nasopalatine canal (NPC) length between the age category was compared using a one-way Analysis of Variance (ANOVA). One-way ANOVA was also used to compared compare the mean nasopalatine canal (NPC) length between the different shapes of NPC as classified by Bahsi et al. [4] and Mardinger et al. [8]. The interaction effect of gender on the mean nasopalatine canal (NPC) length between the different shapes of NPC was analyzed using two-way ANOVA. The *p*-value of less than 0.05 was considered statistically significant.

## 3. Results

A total of 360 CBCT scans were included for data analysis after scrutinizing the eligibility criteria. Table 1 presents the distribution of age based on the gender of the study participants (*N* = 360). Among the participants, 35.83% were male, with a mean age of 36.54 years (SD = 11.25), ranging from 18 to 58 years. The female participants constituted 64.17%, with a slightly higher mean age of 37.24 years (SD = 10.90), within an age range of 18 to 60 years. The overall sample size was 360, with a combined mean age of 36.99 years (SD = 11.01), ranging from 18 to 60 years.

The male participants, constituting 35.83% of the sample, exhibited a mean nasopalatine canal length of 13.58 mm (SD = 3.66), with measurements ranging from 6.5 to 36.03 mm. In contrast, the female participants, representing 64.17% of the total sample, demonstrated a slightly shorter mean nasopalatine canal length of 11.91 mm (SD = 2.72), within a range of 5.78 to 22.34 mm. The overall study cohort, with a combined sample size of 360, showed a mean nasopalatine canal length of 12.51 mm (SD = 3.19), varying between 5.78 and 36.03 mm.

The distribution of nasopalatine canal length (mm) based on the age category of the study participants (*N* = 360) includes 18–30, 31–40, 41–50, and 51–60. Among participants aged 18–30, constituting 35.56% of the sample, the mean nasopalatine canal length was 12.08 mm (SD = 2.75), with measurements ranging from 7.3 to 22.92 mm. In the 31–40 age group (25.56% of the sample), the mean nasopalatine canal length was 12.96 mm (SD = 3.35), varying from 6.04 to 22.34 mm. Participants aged 41–50 (25.83% of the sample) had a mean nasopalatine canal length of 12.53 mm (SD = 3.74), with measurements ranging from 5.78 to 36.03 mm. The 51–60 age category (13.06% of the sample) showed a mean nasopalatine canal length of 12.76 mm (SD = 2.67), ranging from 7.76 to 18.64 mm.

The comparison of nasopalatine canal shapes based on the gender of the study participants (*N* = 360) are detailed in Table 2. The nasopalatine canal shapes include banana, cone, cylindrical, funnel, reverse cone, and hourglass. Among male participants, the majority exhibited the hourglass shape, constituting 80.62% (*N* = 104), while other shapes like banana, cone, cylindrical, funnel, and reverse cone were present in smaller percentages (ranging from 0.78% to 7.75%). The female participants also predominantly displayed the hourglass shape at 87.01% (*N* = 201), with the other shapes occurring at lower frequencies (ranging from 0.87% to 6.06%).

The association between gender and nasopalatine canal length in the study participants (*N* = 360) was assessed using an independent sample *t*-test (Table 3). The analysis aimed to assess potential gender-based differences in nasopalatine canal length. The results indicate a statistically significant difference (*p* < 0.001) in nasopalatine canal length between males and females. Among the male participants (*N* = 129), the mean nasopalatine canal length was 13.58 mm, with a standard deviation of 3.66 mm. In contrast, female participants (*N* = 231) exhibited a mean nasopalatine canal length of 11.91 mm, with a standard deviation of 2.72 mm.

The results of a one-way analysis of variance (ANOVA) conducted to examine the association between age category and nasopalatine canal length among the study participants are shown in Table 4. The analysis aimed to explore whether there were statistically significant differences in nasopalatine canal length across different age categories in NPC. The *p*-value associated with the ANOVA test is 0.217, suggesting no statistically significant difference in mean nasopalatine canal length among the age categories (18–30, 31–40, 41–50, and 51–60). The means and standard deviations for nasopalatine canal length in each age category are as follows: 12.08 mm (2.75) for the 18–30 age group, 12.96 mm (3.35) for the 31–40 age group, 12.53 mm (3.74) for the 41–50 age group, and 12.76 mm (2.67) for the 51–60 age group.

To analyze the association of the nasopalatine canal shape and length among the study participants, a one-way analysis of variance (ANOVA) was conducted (Table 5). The analysis aimed to determine if there are statistically significant differences in nasopalatine canal length across different shapes of the nasopalatine canal. The *p*-value associated with the ANOVA test is 0.076, suggesting no statistically significant difference in mean nasopalatine canal length among the different shapes of NPC.

The results of a two-way analysis of variance (ANOVA) examining the association between nasopalatine canal shape and nasopalatine canal length, with a specific focus on gender-based stratification among the study participants (*N* = 360), are represented in Table 6. The analysis investigates whether there are significant differences in the length of the nasopalatine canal across various shapes, considering the interaction effect with gender. The *p*-value for the interaction effect is 0.046, indicating a statistically significant association, indicating the relationship between nasopalatine canal shape and length is influenced by gender.

## 4. Discussion

This observational study investigated the length and shape of the nasopalatine canal (NPC) using cone-beam computed tomography (CBCT) images from a sample of 360 participants. Prior investigations have employed various imaging techniques to study the NPC, including multislice computed tomography (MSCT) scans [7,8], high-resolution magnetic resonance imaging (MRI) [9], micro-CT scans [10], and cone-beam computed tomography (CBCT) imaging [11,12,13,14]. Notably, CBCT offers a significant advantage over MSCT by delivering a lower radiation dose while providing comparable or superior spatial resolution for detailed imaging. Also, while traditional examinations like lateral cephalometric X-rays can detect the presence of NPC [15], previous research has shown limitations in their ability to fully visualize the canal’s size and position due to the two-dimensional nature of the images [16]. We employed cone-beam computed tomography (CBCT) to address these issues and specifically analyzed sagittal cross-sections to classify NPC shapes using the Bahsi et al. [4] and Mardinger et al. [8] classification system. The findings contribute to our understanding of NPC anatomy, potentially informing surgical procedures in the anterior maxilla.

Studies using CBCT technology have shown that the average length of the NPC can vary widely, ranging from 8.1 mm to 16.3 mm [11,12,13,14,15,16,17,18,19,20,21,22]. These studies’ findings align well with the current study’s findings, where the average NPC length was 12.51 mm with a standard deviation of 3.19 mm, but with a significant range (5.78 mm to 36.03 mm). Another study conducted by Bains et al. [23] showed that, on average, males had a longer canal, measuring 14.69 mm, compared to females, whose average canal length was 12.74 mm. The measurement considered the distance from the anterior wall of the foramen to the anterior nasal spine. Interestingly, a recent study on Indian subpopulations reported a slightly longer average NPC length of 18.63 mm with a standard deviation of 2.35 mm [24]. The observed variations in NPC length could stem from several factors. One possibility is inherent differences between the studied populations, potentially due to variations in race and ethnicity. Additionally, the choice of orthogonal plane used for NPC length measurement might influence the results. It is worth noting that the greater average male NPC length compared to females could be attributable to larger craniofacial dimensions in males, precisely the craniocaudal distance [25].

An interesting finding in this study is a statistically significant difference in NPC length between males and females. Males had a statistically longer NPC (13.58 mm) than females (11.91 mm). These findings were similar to the studies done by Hakbilen and Magat, Jornet et al., and Linjawi et al. [13,18,26] in contrast to the findings of Etoz et al. [27]. This gender difference is essential to consider during surgical procedures in the anterior maxilla, as knowing the expected NPC length can help dentists with anesthesia delivery and avoid potential complications. In contrast to this study, other studies have reported no significant difference in NPC length based on gender [4,19]. 

The study did not find a significant difference in NPC length across the age groups (18–60 years) examined, similar to the studies conducted by Al-Amery et al. [20], Koppera et al. [19], and Thakur et al. [28]. These findings suggest that NPC length reaches its adult size by early adulthood and remains relatively stable throughout adulthood. While Bajoria et al. [29] found statistically significant differences in NPC length across age groups (*p* = 0.0001), they suggested that dental status, rather than age itself, might be the underlying factor. Mardinger et al. [8] support this notion, proposing that the NPC is not static. Their findings indicate that the canal expands in all dimensions after tooth extraction and with age, suggesting that the edentulous group in Bajoria’s [29] study might have been older than the dentate group. Consequently, the observed morphological differences could be due to a combination of factors: the presence or absence of teeth and age-related changes in bone quality and quantity. However, Keskek et al. [6] reported a statistically significant difference between age and NPC length, stating that the shortest NPC lengths were observed among participants between 9 and 18 years of age. Another research by Sudheer et al. [30] suggests a decrease in length with increasing age. This inconsistency might be due to two factors. Firstly, natural bone resorption occurs over time, potentially explaining the reduction in NPC length reported by Sudheer et al. [30]. Secondly, certain systemic conditions like diabetes mellitus and osteoporosis, particularly in postmenopausal women, can alter bone morphology and potentially influence NPC size. These findings highlight the need for further investigation into the influence of various factors, including age, health conditions, and population demographics, on NPC anatomy [24].

The most common NPC shape was hourglass (84.72%), followed by less frequent shapes like a funnel (4.44%), banana (0.83%), cone(5.83%), reverse cone (2.78%), and cylindrical (1.39%). While the overall NPC length did not significantly differ based on shape, the finding that hourglass is the dominant shape provides a helpful reference for dentists when interpreting CBCT images. These results align with the findings by Etoz and Sisman [27], who reported that 38.78% of the NPCs exhibited an hourglass-like shape; funnel-shaped canals were the second most common, representing 27.35% of the total. Less frequent shapes were conical (9.18%) and cylindrical (8.25%). In contrast, Milanovic et al. [5], Lake et al. [31], and Linjawi et al. [13] reported the highest occurrence of funnel-shaped NPCs. Cone-shaped NPCs were commonly observed in the study by Alasmari [19]. At the same time, cylindrical canals were frequently reported by Bahsi et al. [4], Nikkerdar et al. [17], Mardinger et al. [8], Liang et al. [7], and Mishra et al. [32]. Another study by Bajoria et al. [29] found a clear dominance of cylindrical canals, with a prevalence of nearly half (47%) of all nasopalatine canals (NPCs) exhibiting this shape. Funnel-shaped canals were the second most common, representing 42%. Hourglass and spindle shapes were significantly less frequent, making up only 7% and 4% of the observed NPCs. Similarly, Koppera et al. [25] found that cylindrical was the most common NPC shape (64.44%), and hourglass-shaped canals were the least frequent (11.11%) in both males and females. One prior study by Gil-Marques et al. [33] reported a high prevalence of banana-shaped nasopalatine canals (NPCs). These variations in anatomy may stem from differences in age, gender, and race among the sampled individuals and disparities in the measurement techniques utilized across various studies [19]. 

There was evidence of a statistically significant interaction effect between gender and NPC shape on NPC length. The *p*-value for the interaction effect is 0.046, which is a particularly intriguing finding. However, Rai et al. [3] and Etoz and Sisman [27] observed no significant difference in nasopalatine NPC shape based on gender. While the overall analysis did not show a clear association between shape and length, this relationship might be more nuanced and differ between males and females.

This study offers several strengths that contribute to the value of its findings. First, it employed a relatively large sample size of 360 participants. This larger sample size strengthens the generalizability of the results. Generalizability refers to how well the findings of a study can be applied to a broader population. With a larger sample, the results are more likely to reflect the characteristics of the population of interest, rather than just the specific group studied. Second, the study utilized cone-beam computed tomography (CBCT) images for data collection. CBCT scans provide highly accurate three-dimensional measurements, which are crucial for precisely assessing the length and shape of the nasopalatine canal (NPC). This accurate measurement approach adds to the reliability of the study’s findings. Finally, the study incorporated a well-established classification system to categorize the various shapes of the NPC. A standardized classification system ensures consistency and clarity in how the NPC shapes are described and analyzed, allowing for easier comparison with other studies using the same classification. 

Despite its strengths, the study also has some limitations. The observational nature of the study design restricts its ability to establish causal relationships. Observational studies can identify correlations between variables but they cannot definitively prove that one variable causes change in another. Another limitation is that the participants were recruited from a specific geographical area. Hence, the generalizability of the findings to populations from other geographic locations raises concern. People from different backgrounds might have variations in NPC anatomy.

Additionally, the study did not consider the influence of factors like ethnicity or body mass index in its analysis. These characteristics could potentially influence NPC anatomy, and not accounting for them might be another limitation. Another limitation of the study was that a single examiner recorded all measurements. Hence, the results might differ from those studies, with multiple investigators analyzing the same parameters.

Building on the strengths of this study, several future research directions could be explored. One intriguing finding was the interaction effect between gender and NPC shape on NPC length. Further research could delve deeper into this by performing stratified analyses or using regression models to understand how NPC shape influences length differently in males and females. Another valuable area for future research would be investigating the impact of NPC anatomy on surgical outcomes in the anterior maxilla. Understanding how variations in NPC anatomy affects surgical procedures could improve techniques and patient outcomes. Finally, additional participant characteristics like ethnicity or vital statistics, including height, weight, and body mass index, could reveal potential associations with NPC anatomy in future studies to provide a more comprehensive understanding of how various factors influence the size and shape of the nasopalatine canal.

The present study highlights the significance of gender on the length and shape of the nasopalatine canal. The nasopalatine canal is a critical structure, especially when anterior maxillary ridges are resorbed and if an implant is required. The findings of the study will be helpful in pre-surgical implant planning to avoid trauma to the NPC and its contents, thereby circumventing complications or deterring implant placement. The different shapes of the NPC canals and the most common shapes in the study population will provide information during pathological evaluation in the NPC region. Thus, the study of this parameter will be helpful in pre-surgical planning, during surgical exploration of the NPC region, and during post-surgical evaluation.

## 5. Conclusions

This study provides valuable reference data on the length and shape of the NPC using CBCT. The mean nasopalatine canal length was found to be 12.51 mm. The hourglass shape of the nasopalatine canal was the most typical shape for both genders in the study. The nasopalatine shape and length have a significant influence on gender. The patient’s age does not influence the length of the canal. Also, the shape and length of the nasopalatine canal are not significantly different. The findings highlight potential gender differences in NPC length and a complex interplay between NPC shape and gender. Further research is warranted to explore these aspects in greater detail and elucidate the clinical implications for surgical procedures in the anterior maxilla.

## Figures and Tables

**Table 1:** Distribution of age based on the gender of the study participants (*N* = 360).

Gender	Frequency	Percentage	Age		
			Mean (SD)	Minimum	Maximum
Male	129	35.83	36.54 (11.25)	18	58
Female	231	64.17	37.24 (10.90)	18	60
Total	360	100	36.99 (11.01)	18	60

**Table 2:** Comparison of shapes of nasopalatine canal based on the gender of the study participants (*N* = 360).

Nasopalatine Canal Shape	Men *N* (%)	Women *N* (%)	Total *N* (%)
Banana	1 (0.78)	2 (0.87)	3 (0.83)
Cone	7 (5.43)	14 (6.06)	21 (5.83)
Cylindrical	3 (2.33)	2 (0.87)	5 (1.39)
Funnel	10 (7.75)	6 (2.60)	16 (4.44)
Reverse cone	4 (3.10)	6 (2.60)	10 (2.78)
Hourglass	104 (80.62)	201 (87.01)	305 (84.72)

**Table 3:** The study’s association between gender and nasopalatine canal length was measured by an independent sample *t*-test (*N* = 360).

Gender	Frequency (*N*)	Mean (SD)	*p*-Value
Male	129	13.58 (3.66)	<0.001
Female	231	11.91 (2.72)	

**Table 4:** Association between age category and nasopalatine canal length of the study participants by one-way ANOVA (*N* = 360).

Age Category	Frequency (*N*)	Mean (SD)	*p*-Value
18–30	128	12.08 (2.75)	0.217
31–40	92	12.96 (3.35)	
41–50	93	12.53 (3.74)	
51–60	47	12.76 (2.67)	

**Table 5:** Association between NPC shape and nasopalatine canal length of the study participants by one-way ANOVA (*N* = 360).

Nasopalatine Canal Shape	Frequency (*N*)	Mean (SD)	*p*-Value
Banana	3	11.88 (6.05)	0.076
Cone	21	10.74 (2.28)	
Cylindrical	5	10.29 (3.96)	
Funnel	16	12.95 (2.34)	
Reverse cone	10	12.55 (2.21)	
Hourglass	305	12.65 (3.23)	

**Table 6:** The association between NPC shape and nasopalatine canal length was stratified by gender of the study participants by two-way ANOVA (*N* = 360).

Nasopalatine Canal Shape	Men (*N* = 129) Mean (SD)	Women (*N* = 231) Mean (SD)	*p*-Value
Banana	17.88	8.89 (4.39)	0.046
Cone	10.43 (1.75)	10.89 (2.54)	
Cylindrical	11.35 (5.19)	8.7 (0.70)	
Funnel	13.10 (2.15)	12.70 (2.83)	
Reverse cone	13.86 (1.34)	11.68 (2.33)	
Hourglass	13.85 (3.79)	12.03 (2.71)	

## Data Availability

The dataset is available upon request from the authors.
